# Integrating artificial intelligence into a talent management model to increase the work engagement and performance of enterprises

**DOI:** 10.3389/fpsyg.2022.1014434

**Published:** 2022-11-25

**Authors:** Maja Rožman, Dijana Oreški, Polona Tominc

**Affiliations:** ^1^Faculty of Economics and Business, University of Maribor, Maribor, Slovenia; ^2^Faculty of Organization and Informatics, University of Zagreb, Zagreb, Croatia

**Keywords:** artificial intelligence, talent management, employees, employee engagement, performance of the company

## Abstract

The purpose of the paper is to create a multidimensional talent management model with embedded aspects of artificial intelligence in the human resource processes to increase employees' engagement and performance of the enterprise. The research was implemented on a sample of 317 managers/owners in Slovenian enterprises. Multidimensional constructs of the model include several aspects of artificial intelligence implementation in the organization's activities related to human resource management in the field of talent management, especially in the process of acquiring and retaining talented employees, appropriate training and development of employees, organizational culture, leadership, and reducing the workload of employees, employee engagement and performance of the enterprise. The results show that AI supported acquiring and retaining a talented employees, AI supported appropriate training and development of employees, appropriate teams, AI supported organizational culture, AI supported leadership, reducing the workload of employees with AI have a positive effect on performance of the enterprise and employee engagement. The results will help managers or owners create a successful work environment by implementing artificial intelligence in the enterprise, leading to increased employee engagement and performance of the enterprise. Namely, our results contribute to the efficient implementation of artificial intelligence into an enterprise and give owners or top managers a broad insight into the various aspects that must be taken into account in business management in order to increase employee engagement and enterprise’s competitive advantage.

## Introduction

The global COVID-19 has undoubtedly accelerated the process of deploying artificial intelligence. Therefore, developing the capacity to deploy and use artificial intelligence in an enterprise is even more important for its competitiveness (Nayal et al., 2021). The future is primarily related to the advancement of new intelligent technologies and the rapid development of computer capabilities (Dhamija and Bag, 2020). During the Industrial Revolution an essential part of the development of technological innovation and the transformation of many routine tasks and processes, which had existed for decades, was observed, especially when people reached the physical limits of capacity (Dabbous et al., 2022). Artificial intelligence offers a similar transformational potential to increase and possibly relocate human tasks in social, industrial and intellectual fields (Munir et al., 2022). The impact of artificial intelligence technologies can be significant, especially in activities such as finance, human resources, healthcare, manufacturing, retail, supply chain, logistics, and the public sector (Paschen et al., 2019). The need to use artificial intelligence has grown with opportunities for digitization. Processes in enterprises have been shortened, a large part of business communications takes place *via* digital media, and last, but not least, part of the business has moved to digital platforms (Goel et al., 2022). With the advent of internet business, new metrics have also emerged that require in-depth and computationally demanding analytics. The advantage of artificial intelligence for the use of marketing analysis is that it enables the calculation and allocation of large databases and learning. Artificial intelligence works similarly to humans and learns similarly to humans (Danyluk and Buck, 2019; Dabbous et al., 2022).

The advantage of using AI is that the knowledge generated by artificial intelligence becomes an added value to the enterprise. This can protect the employer from leaking knowledge (Goel et al., 2022). The knowledge that an enterprise would acquire in the case of using artificial intelligence in marketing is knowing the customers, their preferences, their behavior, knowledge of the business environment and its changes, and knowledge of the enterprise, strategies and desires (Lauterbach, 2019). In human resource management, talent acquisition, education, and other fundamental areas of human resources, using artificial intelligence affects changes in the work environment and the entire field of human resource management (Kumar et al., 2020; Saxena and Kumar, 2020). New artificial intelligence technologies that help automate HR processes could be the key to solving some of the HR function’s challenges, where less resources could be achieved more (Nayal et al., 2021). AI can reduce the stress of finding a suitable candidate and thereby reduce the monotony of the work of managers in finding candidates with the desired qualifications (Kiron, 2017; Wamba-Taguimdje et al., 2020; Kambur and Akar, 2021).

Increasing the use of digital technologies through artificial intelligence will significantly impact changes in the labor market, namely the focus on individual work tasks and the departure from standard forms of employment (Lee and Chen, 2022). Nowadays, the digitization of business models is one of the biggest challenges in all industries. Digital technologies strongly impact how enterprises create and bring added value to their customers. In addition, enterprises need to update their business models, emphasizing integrating technology into their internal organization, administration, operations, and strategy (Di Francescomarino and Maggi, 2020). Although there is a lot of enthusiasm for the value of artificial intelligence, enterprises starting to use artificial intelligence solutions face a number of challenges that prevent them from achieving greater performance (Bag et al., 2020). There are many challenges in enterprises that managers and employees face when they want to introduce an artificial intelligence system into their work process (Chiarini et al., 2020; Amoako et al., 2021). These limitations range from systems bias and distrust in data collection and algorithms to more theoretical issues related to the decision-making system and taking control of workplace decision-making (Okunlaya et al., 2022). Adopting artificial intelligence is considered a challenge for enterprises. New technologies, such as artificial intelligence, will change the way we work and consequently affect the organization of work and processes in the enterprise (Yigitcanlar et al., 2020). Therefore, the enterprise must adequately implement artificial intelligence systems into existing processes and properly educate employees to avoid feelings of self-preservation and conflict situations (Soni, 2020).

Also, the battle for talented employees has never been so challenging. Due to the automation of repetitive tasks, jobs are changing and some jobs are even disappearing, while the number of very complex tasks is growing. In view of this, modern human resources departments face challenges we have not yet seen (Kambur and Akar, 2021). Artificial intelligence technologies can significantly support finding and hiring employees and improve employees’ well-being and loyalty, and work experience. Artificial intelligence technologies help the enterprise build a competitive advantage with technology and the talents it employs (Wamba-Taguimdje et al., 2020).

AI has an increasing effect on the economy and presents a new dimension of business. Slovenian enterprises need to take a step forward in using artificial intelligence, both in supporting business and production processes and in upgrading the products and services themselves. The implementation of artificial intelligence requires the transformation of the entire enterprise, which manifests itself through organizational culture, new management methods, new employee training methods and the creation of new enterprise strategies. The problem of implementing artificial intelligence is also manifested in the fact that enterprises should change the way their employees work. The goal of artificial intelligence is to optimize, automate, or offer decision support in the enterprise. Artificial intelligence increases productivity, and pave the way for new products. All this can lead to a change in the way people do their work. Thus, enterprises will need to carefully analyze expected outcomes and prepare plans to adjust their workforce capabilities, priorities, goals and jobs accordingly. Thus, managing artificial intelligence models requires new types of work skills. Based on this, we developed a multidimensional model with key constructs that are important in implementing artificial intelligence in the enterprise to increase employee engagement and performance of the enterprise (Fountaine et al., 2019). Also, we formulated two research questions: (1) Are there positive effect of key constructs that are important in implementing artificial intelligence in the enterprise (AI supported acquiring and retaining a talented employees, AI supported appropriate training and development of employees, appropriate teams, AI supported organizational culture, AI supported leadership, reducing the workload of employees with AI; Shanmugam, 2015; IBM, 2018; Hunt, 2021) on performance of the enterprise? and (2) are there positive effect of key constructs that are important in implementing artificial intelligence in the enterprise (AI supported acquiring and retaining a talented employees, AI supported appropriate training and development of employees, appropriate teams, AI supported organizational culture, AI supported leadership, reducing the workload of employees with AI) on employee engagement?

## Literature review

### Definition of talent management and talent employees

The term talent management is composed of the word talent, which means a personal ability (mental or physical), which makes one person stand out from the crowd of others, and the word management, which means managing, leading and dealing with individuals (Narain et al., 2019; Jooss et al., 2022; Kafetzopoulos et al., 2022). Thus, talent management is an effective way of managing individuals who are very successful in their field of operation in the enterprise (Aljbour et al., 2021). The term talent is associated with the ability to find the cause of a problem, synthesize information and create solutions or ways to solve a certain problem (Luna-Arocas et al., 2020). Talent is one of the most popular characteristics that employers expect from their employees, which enables them to perform better than average employees. The characteristics of talented employees are shown in the fact that they are curious, set ambitious goals, like to do several things at the same time, and work long and hard on things that actually interest them (Costello and Osborne, 2005; Mensah, 2015). Talent management is a strategic approach to business planning and human resources management, as well as one of the new ways of achieving organizational efficiency (Kafetzopoulos et al., 2022). Such an approach makes it possible to improve the results and potential of human resources (specifically – talents), which can bring a measurable and essential difference to the enterprise (Aljbour et al., 2021). Talent management is the answer to the challenges of attracting and retaining employees with high competences and enabling those employees to achieve extraordinary work results, develop and advance in the enterprise (King, 2017). Schreuder and Noorman (2019) define talent management as placing the right employees with the right skills in the right position in the enterprise. Talent management includes three key activities, which are talent acquisition, talent development and talent retention (Seethalakshmi et al., 2020; Jooss et al., 2022). The retention and development of talent at the individual level are important components of the talent management strategy (Pandita and Ray, 2018). The retention of key talents in the enterprise must be supported through various human resources management activities, which must be additionally adapted to the talents (Yildiz and Esmer, 2022). Two of the ways to realize this are the development of individual career development plans and the development of programs that encourage employees to grow together with the enterprise (Kafetzopoulos et al., 2022). Talent management activities must be aimed at increasing employee engagement, because this directly affects the possibilities for long-term talent retention in the enterprise and the achievement of the enterprise’s performance (Pandita and Ray, 2018; Yildiz and Esmer, 2022).

### Definition of AI

Artificial intelligence mimics the ability of the human brain to learn, analyze, and make decisions (Mikalef and Gupta, 2021). Jabłońska and Pólkowski (2017) emphasize that the key reasons for implementing artificial intelligence-based processes include problem-solving, reducing the human workload, and reducing the cost of cheaper labor. Thus, artificial intelligence represents the next step in developing enterprise, where employers can now capture, store and analyze more data and information than ever before. Adapting, investing, and conducting research and development of advanced systems is increasing rapidly on the part of both the state and enterprises around the world (Davenport and Ronanki, 2018; Eubanks, 2018). In addition, according to Mikalef and Gupta (2021), artificial intelligence can foster creativity in enterprises. Automating many repetitive and manual tasks will allow employees to have more time for creative activities. Also, with certain applications, artificial intelligence can increase employees’ ability to perform tasks with the help of extended intelligence (Eubanks, 2018). Special artificial intelligence techniques can manage a large set of data and help professionals with creative tasks such as engineering by improving their input and making suggestions that would otherwise be difficult to develop (Bag et al., 2020; Yigitcanlar et al., 2020).

### Implementation of artificial intelligence in an enterprise

The biggest challenge in implementing artificial intelligence is changing the enterprise’s culture and leadership, acquiring new knowledge and skills, and changing business processes (Eriksson et al., 2020). AI in the field of HRM in the enterprise means using technology to solve tasks in various human resources processes, especially in the field of talent acquisition, education, employee development, and workforce management (Kambur and Akar, 2021). Therefore, the enterprise must take care of employees’ proper training and development (Eriksson et al., 2020). AI can be used in practically all phases of work in human resource management, from short-term talent selection and candidate screening to subsequent procedures for introducing new employees and evaluating performance (Mikalef and Gupta, 2021). In addition to restructuring the repetitive administrative tasks, artificial intelligence tools help to rationalize personnel tasks and gain exceptional insights into each candidate and employee (Di Francescomarino and Maggi, 2020). Artificial intelligence tools for human resource management represent the future of work, as they perform their tasks without the limitations of human bias and the ability to make mistakes (Coulibaly et al., 2019). In general, artificial intelligence is mainly used in high-tech enterprises, the financial industry, healthcare, and logistics. In Slovenia, the biggest lag can be felt in processing and manufacturing enterprises, agriculture, healthcare, tourism, and trade (SURS, 2021a). Organization and human resources are the biggest problem in enterprises. Most enterprises lag far behind in their readiness to introduce artificial intelligence. Enterprises do not have a sufficient number of employees with the appropriate skills. The prevailing belief in most enterprises is that they need technical personnel who know how to develop and maintain technologies. This is the biggest mistake made by most Slovenian enterprises (SURS, 2021a). There are two equivalent branches of necessary knowledge and innovation when introducing artificial intelligence. In addition to technology, organizational, process, and personnel aspects are also important. Enterprises need employees with the skills to connect business and technology. Above all, enterprises need employees who know how to recognize the opportunities offered by artificial intelligence and how to use it in their work. Therefore, enterprises need to invest heavily in the education, training and retraining of all employees (Shaffer et al., 2020). These are the biggest backlogs of Slovenian enterprises (SURS, 2020). On the other hand, there are also risks that affect the quality of decision-making by leaders, considering the results of data analysis using artificial intelligence. AI algorithms are made by humans, they can have built-in biases from those they inadvertently or intentionally introduce into the algorithm. In the event that artificial intelligence algorithms are built biased, they will produce biased results (Tambe et al., 2019; Paesano, 2021). AI may be that some important aspects are not included in the algorithm or that it is programmed to reflect and reproduce a structural bias. Moreover, bias during decision-making can be attached not only to human decision-making, but also to decisions made by artificial intelligence, since bias can already appear during machine learning (Barn, 2020; Pangarso et al., 2022).

The first step towards expanding the use of AI among enterprises is to raise the level of awareness of how AI will have a positive impact on their business in the future (SURS, 2021b). Also, the use of artificial intelligence today is no longer limited to large enterprises. Due to the availability of technologies, smaller enterprises can also use them to improve their business (Wamba-Taguimdje et al., 2020). Thus, the enterprise must have a comprehensive strategy for implementing artificial intelligence (Yigitcanlar et al., 2020). Therefore, we designed essential constructs that are crucial in implementing artificial intelligence in the enterprise, increasing employee engagement and performance of the enterprise.

#### Acquiring and retaining a talented employees

AI helps analyze the profiles of different candidates, where it checks whether the candidates have the required competencies. It also helps with communication by sending automated emails to candidates. With the help of artistic intelligence, employers get an in-depth set of required knowledge and skills, thus helping to select potential employees in acquire talent in a much faster time (Vaishnavi and Achwani, 2018). Technology helps HR professionals select suitable candidates for the job and allows them to devote more time to tasks with greater added value and focus on more critical parts of the business and strategic tasks (Eubanks, 2018; Hogg, 2019). Talented employees can connect and structure business processes as a whole, know how to solve problems quickly and efficiently, are eager for new challenges, are motivated and self-initiative, confident, curious, capable of empathy, and want to improve business change. Talented employees show great loyalty to the enterprise as they identify with it (Anlesinya and Amponsah-Tawiah, 2020). Thus, we formulated hypotheses:

*H1*: AI supported acquiring and retaining talented employees have an effect on performance of the enterprise.

*H2*: AI supported acquiring and retaining talented employees have an effect on employee engagement.

#### Appropriate training of employees

Despite the advantages that artificial intelligence offers for performing mentally demanding work, the evaluation of an investment in artificial intelligence needs to be evaluated appropriately (Goel et al., 2022). An enterprise may start programming artificial intelligence, but it gets stuck in transferring employees’ tacit knowledge into a programming language (De Bruyn et al., 2020). Employees do not understand different phenomena independently, which makes it difficult to transfer certain decisions made in the business world to artificial intelligence, which will otherwise perform all operations rationally (Kambur and Akar, 2021). Another problem is the transfer of knowledge in the opposite direction. The knowledge created by artificial intelligence needs to be transferred to employees, which is an even greater challenge, as the data needs to be presented in a visual form that will facilitate the transfer of knowledge. In addition, the learning cycle needs to be repeated for employees, as with artificial intelligence (Maity, 2019; De Bruyn et al., 2020). Artificial intelligence can also be used to smooth learning and development activities. For example, an enterprise can use artificial intelligence to develop a custom learning program for its employees (Soltani et al., 2020). This program can be tailored to the individual’s needs and preferences, which will help them learn new skills more quickly and effectively (Maity, 2019). Thus, artificial intelligence improves employees’ engagement levels and helps them learn faster (Kashive et al., 2021). Additionally, enterprises can use artificial intelligence to track employee progress and provide feedback accordingly (Paesano, 2021). This will help employees feel more supported, motivated, and engaged as they develop their skills (Wijayati et al., 2022). The following hypotheses were formulated:

*H3*: AI supported appropriate training and development of employees have an effect on performance of the enterprise.

*H4*: AI supported appropriate training and development of employees have an effect on employee engagement.

#### Forming appropriate teams

New ideas, new views on production or products, and how employees work bring enterprises opportunities to compete more successfully. Each team can develop creative processes for themselves that help them improve efficiency and better solve tasks, leading to an increase in employees’ work engagement (Webber et al., 2019). With the help of artificial intelligence, employees can now collaborate with teams quickly and easy. The technology can identify and group similar topics, which makes it easier for team members to work on specific tasks related to a project. Also, it helps reduce misunderstandings and strengthens relationships between employees (Arslan et al., 2021). Artificial intelligence can help employees communicate more efficiently by automatically sorting and organizing incoming emails, messages, and documents. Also, it can provide summaries of conversations or specific topics to help employees stay up-to-date on all the latest developments. Consequently, employees will spend less time managing communications and more time working on tasks (Saxena and Kumar, 2020). Artificial intelligence is used as a communication tool for enterprises with employees working from home or in different locations. This allows them to communicate and update the necessary information about their work or projects they are working on (Lesgold, 2019). Therefore, it is a necessity that leaders facilitate and build the teamwork skills of their employees if they are to steer an enterprise toward success (Webber et al., 2019). The following hypotheses were formulated:

*H5*: Appropriate teams have an effect on performance of the enterprise.

*H6*: Appropriate teams have an effect on employee engagement.

#### New organizational culture

For an enterprise to be ready for the future, its leaders need to create an innovative organizational culture. Organizational culture is key to building an artificial intelligence-driven enterprise (Munir et al., 2022). Enterprises that manage to build a positive artificial intelligence culture and an inclusive and inspiring environment will successfully manage change and attract all their employees (Behl et al., 2021). The leader must create a culture that will allow the enterprise to develop and adapt to new business realities quickly. This will be expressed through better ideas and products and will help create a more inclusive future (Jarrahi et al., 2022).

Moreover, artificial intelligence chatbots help enterprises engage their employees and maintain a positive and inclusive work culture regardless of background. This helps bring people together by creating an open environment for all employees, not just those closest to senior management (Dabbous et al., 2022). It is important to create a new organizational culture that encourages experimentation and continuous innovation, and the development of new solutions. This in turn leads to increased performance of the enterprise (Behl et al., 2021). Building a culture that supports innovation with artificial intelligence affects competitiveness. Based on a global survey of 2,197 managers, 75% of respondents saw improvements in team morale and engagement, collaboration, and collective learning (Ransbotham et al., 2021). Thus, we proposed hypotheses:

*H7*: AI supported organizational culture has an effect on performance of the enterprise.

*H8*: AI supported organizational culture has an effect on employee engagement.

#### New ways of leadership

One of the main obstacles to adopting artificial intelligence is the lack of leadership support for artificial intelligence initiatives. Realizing the business value of investing in artificial intelligence requires leaders’ genuine understanding and commitment to drive far-reaching change (Mikalef and Gupta, 2021). The implementation of artificial intelligence in the enterprise will be maximized because of the role of a leader (Dhamija et al., 2021). New technologies like artificial intelligence have changed the nature of leadership. The use of robust data analytics grounded in artificial intelligence and machine learning techniques reveals new business applications insights (Wijayati et al., 2022). With the use of artificial intelligence, leaders will focus more on the human aspects (for example, personality characteristics and behaviors) and less on the cognitive processing of facts and information (Chang, 2020). This will help improve employee engagement and performance and increase operational efficiencies to improve the enterprise’s bottom line (Kambur and Akar, 2021). Thus, we proposed hypotheses:

*H9*: AI supported leadership has an effect on performance of the enterprise.

*H10*: AI supported leadership has an effect on employee engagement.

#### Reducing the workload of employees with artificial intelligence

Today, the introduction of AI in business human resources processes requires a new symbiosis between human resources, technology, and employees, as it affects the enterprise’s work tasks and business processes (Dhamija and Bag, 2020). Artificial intelligence will increasingly perform routine operational tasks, thus enabling employees to devote their time to more creative and strategic tasks that help develop the human resources function of the future (Goel et al., 2022; Lee and Chen, 2022). Artificial intelligence influences employee engagement by improving remote employee monitoring. With artificial intelligence-powered tools, employees can now collaborate more quickly and effectively with colleagues who are located remotely. This helps to enhance communication and collaboration between employees (or team members), regardless of their location (Agarwal et al., 2022). Artificial intelligence can help employees set goals, provide timely feedback on their progress, and help them find training courses that are relevant for improving specific skills (Sari et al., 2020). Artificial intelligence allows employees to save nearly a third of their otherwise spent on uncomplicated and monotonous tasks. This leads to an increase in employees’ work performance and employee engagement (Wijayati et al., 2022). According to Bushweller (2020) and Wang (2021), artificial intelligence could help employees in repetitive and time-consuming tasks, which, in turn, would reduce their workload and increase their productivity. Thus, artificial intelligence significantly decreases workplace stress and workload (Dhamija and Bag, 2020; Saxena and Kumar, 2020; Okhifun, 2022). The following hypotheses were formulated:

*H11*: Reducing the workload of employees with AI has an effect on performance of the enterprise.

*H12*: Reducing the workload of employees with AI has an effect on employee engagement.

### Increasing employee engagement and performance of the enterprise with artificial intelligence

Today, artificial intelligence offers excellent value in a market where people are developing artificial intelligence systems to perform complex tasks (Goel et al., 2022). New artificial intelligence applications herald a major step in technology development (Lee and Chen, 2022). Traditional software is powerful but requires a large configuration and setup to provide added value (Cichosz et al., 2020). Artificial intelligence systems are flexible and require less time to complete a particular task, as they learn quickly (Nayal et al., 2021). Nowadays, artificial intelligence is becoming a competitive advantage for early users (Bag et al., 2020). Those enterprises that do not adopt and implement artificial intelligence in their processes will be less competitive and less successful in the market (Okunlaya et al., 2022). Thus, artificial intelligence positively influences performance of the enterprise. The primary goal of implementing artificial intelligence into enterprises’ work processes is to reduce costs and improve the quality of products and services. The use of artificial intelligence encourages enterprises to both innovative and successful responses to modern challenges as well as to improve work by reducing the number of repetitive tasks through automation (Ribeiro et al., 2021). In addition, artificial intelligence with algorithms and techniques enhances the accuracy of the implementation of automated processes (Yigitcanlar et al., 2020). Industry 4.0 is characterized by a set of technologies that enable even greater progress in processes, and automation contributes to the better efficiency of organizational processes and presents new opportunities in the market (Malik et al., 2021; Mikalef and Gupta, 2021). The combination of concepts and technologies such as automation, smart appliances, and processes brings significant changes in business processes, affecting the flow of digital processes throughout the enterprise (Ribeiro et al., 2021). With new technologies, the enterprise can streamline and optimize business processes, relieve employees’ workload, and thus enable faster, more efficient, and higher quality achievement of business goals and results (Eriksson et al., 2020; Yigitcanlar et al., 2020). Bag et al. (2020); Kambur and Akar (2021); Goel et al. (2022) emphasize that enterprises often face a problem when employees lose their potential and creativity in the routine. Artificial intelligence can take over cyclical processes and execute them strictly on schedule. In this way, the enterprise enables employees to have more time for creativity and innovation. In the long term, artificial intelligence can significantly increase the efficiency of the department and the enterprise as a whole (Bag et al., 2020; Kambur and Akar, 2021; Goel et al., 2022). Also, working with large amounts of information is a laborious process that requires a lot of time and resources. Such a task is extremely difficult for humans but easy for artificial intelligence. The use of technology significantly reduces the lead time and eliminates errors (Bushweller, 2020; Sari et al., 2020; Wang, 2021). Thus, we proposed hypotheses:

*H13*: Employee engagement has an effect on performance of the enterprise.

**Figure 1:**
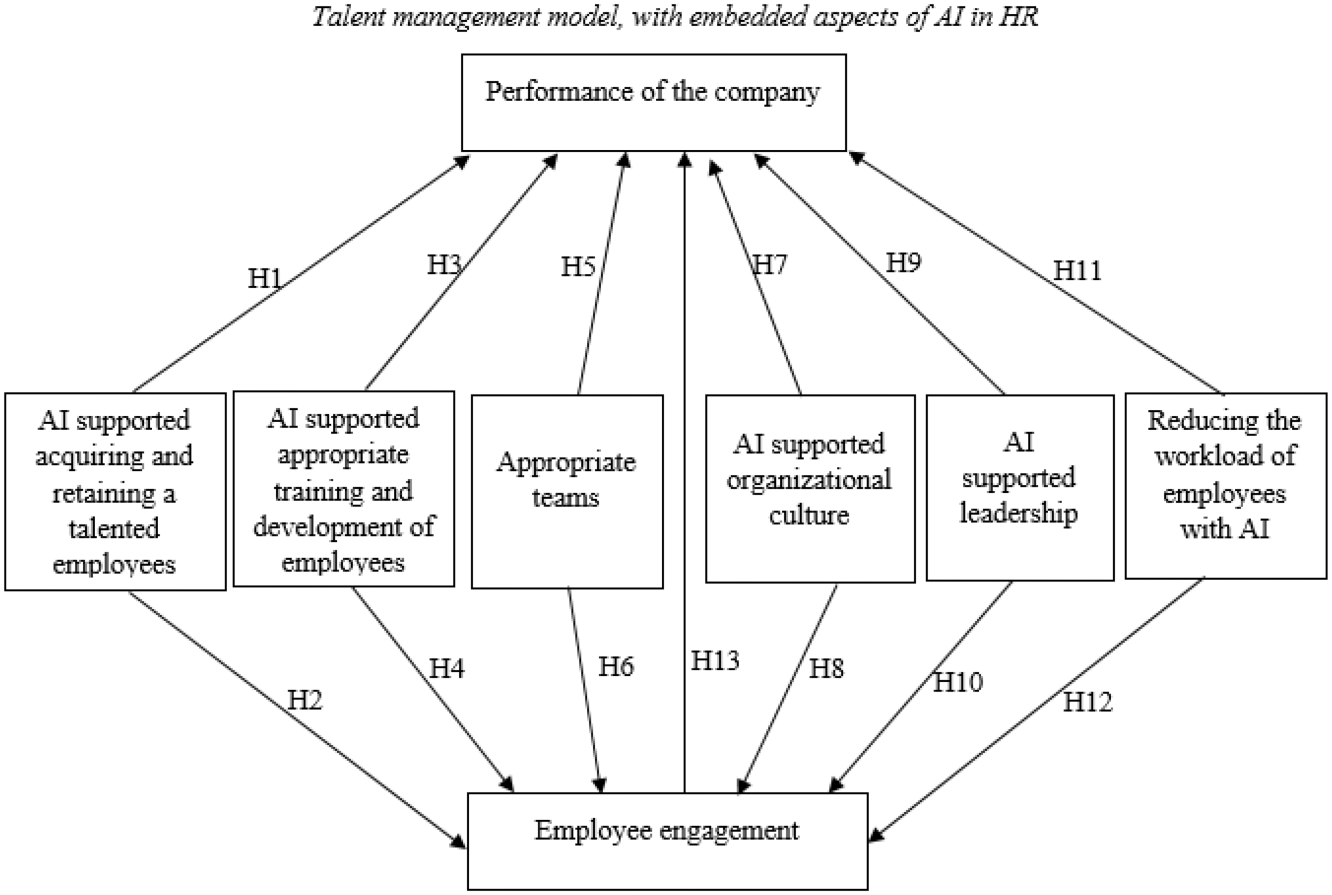
Conceptual model and hypotheses.

Figure 1 presents the conceptual model of implementation of AI in the enterprise to increase employee engagement and performance of the enterprise. Figure 1 shows six independent variables which are AI supported acquiring and retaining a talented employees, AI supported appropriate training and development of employees, appropriate teams, AI supported organizational culture, AI supported leadership, reducing the workload of employees with AI and two dependent variables which are performance of the enterprise and employee engagement.

## Methodology

### Data and sample

The main survey involved randomly selected 317 medium-sized and large Slovenian enterprises. From each enterprise, a manager or owner participated in our research. Data were collected from April 2022 to the end of June 2022. Random sampling was carried out from the population, where the sample frame is represented by the AJPES (Slovenian Business Register) database of business subjects (AJPES, 2022). Empirical research was conducted in 500 randomly selected medium-sized and large enterprises out of 2,576 medium-sized and large Slovenian enterprises (AJPES, 2022). In research, the main survey involved 317 medium-sized and large enterprises. The response rate was 63.4%. When considering non-responses in the questionnaire, we took into account the non-response of the element and the non-response of the variable. Eight enterprises did not answer the questionnaire, so we excluded them from consideration, 317 medium-sized and large enterprises answered the questionnaire in full. According to the Companies Act (ZGD-1, 2022), for medium-sized enterprises, the average number of employees in a business year does not exceed 250, while for large enterprises, the average number of employees in a business year exceeds 250 employees. According to the standard classification of enterprises activities, managers or owners were from manufacturing (25.9%), trade, maintenance, and repair of motor vehicles (23.9%), information and communication activities (22.4%), financial and insurance activities (18.6%), professional, scientific and technical activities (7.9%) and other diversified business activities (1.3%). The biggest share of enterprises presents large enterprises (54.6%). Medium-sized enterprises comprised 45.4%. According to gender, 57.1% of male and 42.9% of female respondents participated in the study.

### Research instrument

We used a questionnaire which was closed type a 5-point Likert-type scale. The questionnaire was translated into the Slovenian language. Items for construct AI supported acquiring and retaining a talented employees were adopted from Kambur and Akar (2021). The items for construct AI supported acquiring and retaining a talented employees referred to the usefulness of AI in acquiring and retaining a talented employees and which skills are required for employment. Items for construct AI supported appropriate training and development of employees were adopted from Pillai and Sivathanu (2020) and referred to the usefulness of AI in training and development of employees. Items for construct appropriate teams were adopted from Mikalef and Gupta (2021). Items for construct appropriate teams refer to the work of team members. An enterprise that uses artificial intelligence technology should have a well-formed team where team members produce many novels and valuable ideas, they work without a leader, solve problems independently, etc. Items for construct AI supported organizational culture were adopted from Dabbous et al. (2022) and relate to whether the organizational culture supports changes and artificial intelligence. Items for construct reducing the workload of employees with AI were adopted from Qiu et al. (2022) refer to whether the enterprise reduces employee stress with the help of AI. Items for constructs AI supported leadership, employee engagement and performance of the enterprise were adopted from Wijayati et al. (2022). Items for construct AI supported leadership relate to understanding business problems and to direct AI initiatives to solve them, design AI solutions to support customers needs, open communiacation, etc. Items for construct employee engagement relate to the way employees are engaged in their work. All items are presented in Table 1.

**Table 1:** Items for each construct.

Construct	Item
AI supported acquiring and retaining a talented employees	AR1: AI helps in a better quality of decisions for recruiting and selecting candidates.
	AR2: AI helps in conducting primary interviews of bulk candidates using chatbots.
	AR3: AI technology save the monotony of the job done during the process of finding candidates.
	AR4: AI technology reduce the time spent in finding candidates.
	AR5: We hire those candidates that have the right skills to accomplish their work successfully.
	AR6: We hire those candidates that are very capable of using ai technologies (e.g., machine learning, natural language processing, deep learning).
	AR7: We hire those candidates that are effective in data analysis, processing, and security.
	AR8: We take care of retaining suitable candidates with help to acquire the necessary skills for their career plan.
AI supported appropriate training and development of employees	TD1: AI technology reduces the time spent on in-enterprise training courses.
	TD2: AI technology reduces the attention deficit that employee experienced in classical in-enterprise training courses processes.
	TD3: AI technology increases accessibility to in-enterprise training courses.
	TD4: In-enterprise training courses with artificial intelligence technology lead to a successful training program.
	TD5: Employee professional knowledge will be kept up to date with in-enterprise training courses through artificial intelligence technology.
	TD6: when the in-enterprise training courses take place with artificial intelligence technology, the restrictions regarding to place where the training will be given will be removed.
	TD7: Employees are provided with the required training to deal with AI applications.
Appropriate teams	AT1: The team members produce many novels and valuable ideas (services/products).
	AT2: The team members work without a leader.
	AT3: The team members coordinate the work themselves.
	AT4: Team members solve problems independently.
	AT5: All team members work equally creatively and enthusiastically to find ideas and solve problems.
	AT6: All team members achieve their goals equally effectively.
AI supported organizational culture	OC1: The enterprise’s culture is very responsive and changes easily.
	OC2: We used AI technology in any part of our business.
	OC3: There is a high level of agreement about how we do things in the enterprise.
	OC4: There is a shared vision of what enterprise will be like in the future.
	OC5: Policies of the enterprise are clearly defined.
	OC6: Employees fully understand the goals of our enterprise.
	OC7: The enterprise’s management provides information to employees in a timely manner.
	OC8: Employees are familiar with all the services / products we offer / produce in our enterprise.
AI supported leadership	L1: We developed a clear vision for what was going to be achieved by our department.
	L2: We are able to understand business problems and to direct AI initiatives to solve them.
	L3: We are able to anticipate future business needs of functional managers, suppliers and customers and proactively design AI solutions to support these needs.
	L4: We are able to work with data scientists, other employees and customers to determine opportunities that AI might bring to our organization.
	L5: Employees have strong leadership to support AI initiatives and are commitment to AI projects.
	L6: In the enterprise prevails open communication and we solve employees’ problems on the spot.
Reducing the workload of employees with AI	RW1: With AI we reduce the burden on administrative staff in the enterprise.
	RW2: The AI technology applied in our enterprise can take orders and complete tasks which reduces the workload of employees.
	RW3: The AI technology applied in our enterprise can communicate with users/customers which reduces the workload of employees.
	RW4: The AI technology applied in our enterprise can search and analyze information which reduces the workload of employees.
	RW5: Artificial intelligence can help in getting the job done which saves employees work time.
Employee engagement	EE1: Using AI enhance employee effectiveness.
	EE2: Employees are engaged to the quality of their work.
	EE3: Employees do their work with passion.
	EE4: Employees are engaged to achieve successful business results.
	EE5: Employees are aware of the importance of innovation for our enterprise and they are helping to develop the enterprise.
	EE6: Employees are enthusiastic in their work.
	EE7: Employees are engaged for business ideas and solutions.
	EE8: Employees believe in the successful development and operation of our enterprise.
Performance of the company	PC1: Through AI the enterprise can able to get accurate results.
	PC2: Through AI the chance of employees error at work are less.
	PC3: AI improves the effectiveness of decisions and actions.
	PC4: AI accelerates making quick and better decisions to achieve successful results.
	PC5: AI provides accurate data and information.
	PC6: Products or services meet the expectations of customers.
	PC7: The delivery of goods or services is conducted in a timely fashion.
	PC8: Compared to our key competitors, our enterprise is growing faster.
	PC9: Compared to our key competitors, our enterprise is more profitable.
	PC10: Compared to our key competitors, our enterprise is more innovative.

AI, artificial intelligence.

### Statistical analysis

We tested the hypotheses with the SEM and used the software tool WarpPLS 7.0. The WarpPLS 7.0 program was used to verify the existence of effects between constructs. We decided to use WarpPLS 7.0 program because it offers many advantages and unique solutions compared to others. We see one of the key advantages in the possibility of explicitly defining non-linear connections between pairs of latent variables (Kock, 2019). As part of the validity, we examined the AVE and CR (Kock, 2019). To check for multicollinearity, we used VIF (Hair et al., 2010). We also used the criterion of quality indicators listed in Table 2 to test the model.

**Table 2:** Factor analysis results.

Construct	Item	Communalities	Loadings	Cronbach’s alpha
AI supported acquiring and retaining a talented employees	AR1	0.742	0.861	0.918
	AR2	0.702	0.838	
	AR3	0.594	0.772	
	AR4	0.590	0.768	
	AR5	0.770	0.878	
	AR6	0.763	0.870	
	AR7	0.765	0.873	
	AR8	0.754	0.867	
KMO = 0.908; Bartlett’s Test of Sphericity: Approx. Chi-Square = 2318.471, df = 28, *p* < 0.001 Cumulative percentage of explained variance: 68.345%				
AI supported appropriate training and development of employees	TD1	0.708	0.841	0.897
	TD2	0.847	0.912	
	TD3	0.838	0.906	
	TD4	0.726	0.869	
	TD5	0.734	0.875	
	TD6	0.845	0.897	
	TD7	0.849	0.908	
KMO = 0.928; Bartlett’s Test of Sphericity: Approx. Chi-Square = 1671.946, df = 21, *p* < 0.001 Cumulative percentage of explained variance: 71.324%				
Appropriate teams	AT1	0.774	0.867	0.889
	AT2	0.741	0.853	
	AT3	0.738	0.849	
	AT4	0.711	0.827	
	AT5	0.748	0.858	
	AT6	0.763	0.869	
KMO = 0.895; Bartlett’s Test of Sphericity: Approx. Chi-Square = 1421.645, df = 15, *p* < 0.001 Cumulative percentage of explained variance: 73.492%				
AI supported organizational culture	OC1	0.726	0.839	0.869
	OC2	0.711	0.843	
	OC3	0.694	0.802	
	OC4	0.673	0.818	
	OC5	0.823	0.904	
	OC6	0.772	0.896	
	OC7	0.706	0.824	
	OC8	0.718	0.851	
KMO = 0.872; Bartlett’s Test of Sphericity: Approx. Chi-Square = 1362.285, df = 28, *p* < 0.001 Cumulative percentage of explained variance: 67.592%				
AI supported leadership	L1	0.874	0.922	0.876
	L2	0.774	0.878	
	L3	0.858	0.916	
	L4	0.708	0.834	
	L5	0.861	0.920	
	L6	0.765	0.866	
KMO = 0.884; Bartlett’s Test of Sphericity: Approx. Chi-Square = 1572.285, df = 15, *p* < 0.001 Cumulative percentage of explained variance: 75.289%				
Reducing the workload of employees with AI	RW1	0.724	0.851	0.940
	RW2	0.683	0.826	
	RW3	0.699	0.836	
	RW4	0.694	0.833	
	RW5	0.638	0.799	
KMO = 0.918; Bartlett’s Test of Sphericity: Approx. Chi-Square = 3275.217, df = 10, *p* < 0.001 Cumulative percentage of explained variance: 72.178%				
Employee engagement	EE1	0.860	0.927	0.948
	EE2	0.828	0.910	
	EE3	0.851	0.922	
	EE4	0.714	0.837	
	EE5	0.732	0.856	
	EE6	0.728	0.853	
	EE7	0.806	0.898	
	EE8	0.706	0.728	
KMO = 0.925; Bartlett’s Test of Sphericity: Approx. Chi-Square = 3062.092, df = 28, *p* < 0.001 Cumulative percentage of explained variance: 74.425%				
Performance of the company	PC1	0.775	0.880	0.948
	PC2	0.825	0.908	
	PC3	0.669	0.836	
	PC4	0.879	0.942	
	PC5	0.806	0.898	
	PC6	0.837	0.915	
	PC7	0.874	0.939	
	PC8	0.765	0.875	
	PC9	0.893	0.952	
	PC10	0.881	0.946	
KMO = 0.938; Bartlett’s Test of Sphericity: Approx. Chi-Square = 5203.703, df = 45, *p* < 0.001. Cumulative percentage of explained variance: 78.576%				

## Research results

In addition to the results in Table 2, the total variance explained for acquiring and retaining a talented employees is 68.345%, for appropriate training and development of employees is 71.324%, for appropriate teams is 73.492%, for organizational culture is 67.592%, for leadership is 75.289%, for reducing the workload of employees is 72.178%, for employee engagement is 74.425% and for performance of the enterprise is 78.576%.

Table 3 shows key quality assessment indicators of the research model.

**Table 3:** Model fit and quality indicators.

Quality indicators	The criterion of quality indicators	Calculated values of indicators of model
Average path coefficient (APC)	p < 0.05	0.528, *p* < 0.001
Average R-squared (ARS)	p < 0.05	0.631, p < 0.001
Average adjusted R-squared (AARS)	p < 0.05	0.674, p < 0.001
Average block variance inflation factor (AVIF)	AVIF <5.0	1.339
Average full collinearity VIF (AFVIF)	AFVIF <5.0	1.482
Goodness-of-fit (GoF)	GoF ≥ 0.1 (low) GoF ≥ 0.25 (medium) GoF ≥ 0.36 (high)	0.624
Simpson’s paradox ratio (SPR)	SPR ≥ 0.7	1.000
R-squared contribution ratio (RSCR)	RSCR ≥0.9	1.000
Statistical suppression ratio (SSR)	SSR ≥ 0.7	1.000
Nonlinear causality direction ratio (NLBCD)	NLBCD ≥0.7	1.000

Table 3 shows that all indicators are suitable. The result of indicator GoF shows that the model is highly appropriate. Table 4 presents indicators of the quality of the structural model.

**Table 4:** Indicators of quality of the structural model.

Constructs	CR	AVE	R^2^	Adj. R^2^	Q^2^	VIF
AI supported acquiring and retaining a talented employees	0.936	0.684	(−)	(−)	(−)	1.161
AI supported appropriate training and development of employees	0.952	0.738	(−)	(−)	(−)	2.296
Appropriate teams	0.944	0.707	(−)	(−)	(−)	2.301
AI supported organizational culture	0.926	0.715	(−)	(−)	(−)	1.076
AI supported leadership	0.958	0.740	(−)	(−)	(−)	1.865
Reducing the workload of employees with AI	0.962	0.826	(−)	(−)	(−)	1.185
Employee engagement	0.952	0.869	0.473	0.458	0.379	1.071
Performance of the company	0.970	0.865	0.469	0.450	0.326	1.042

(−) values cannot be calculated because the construct is a baseline.

**Figure 2:**
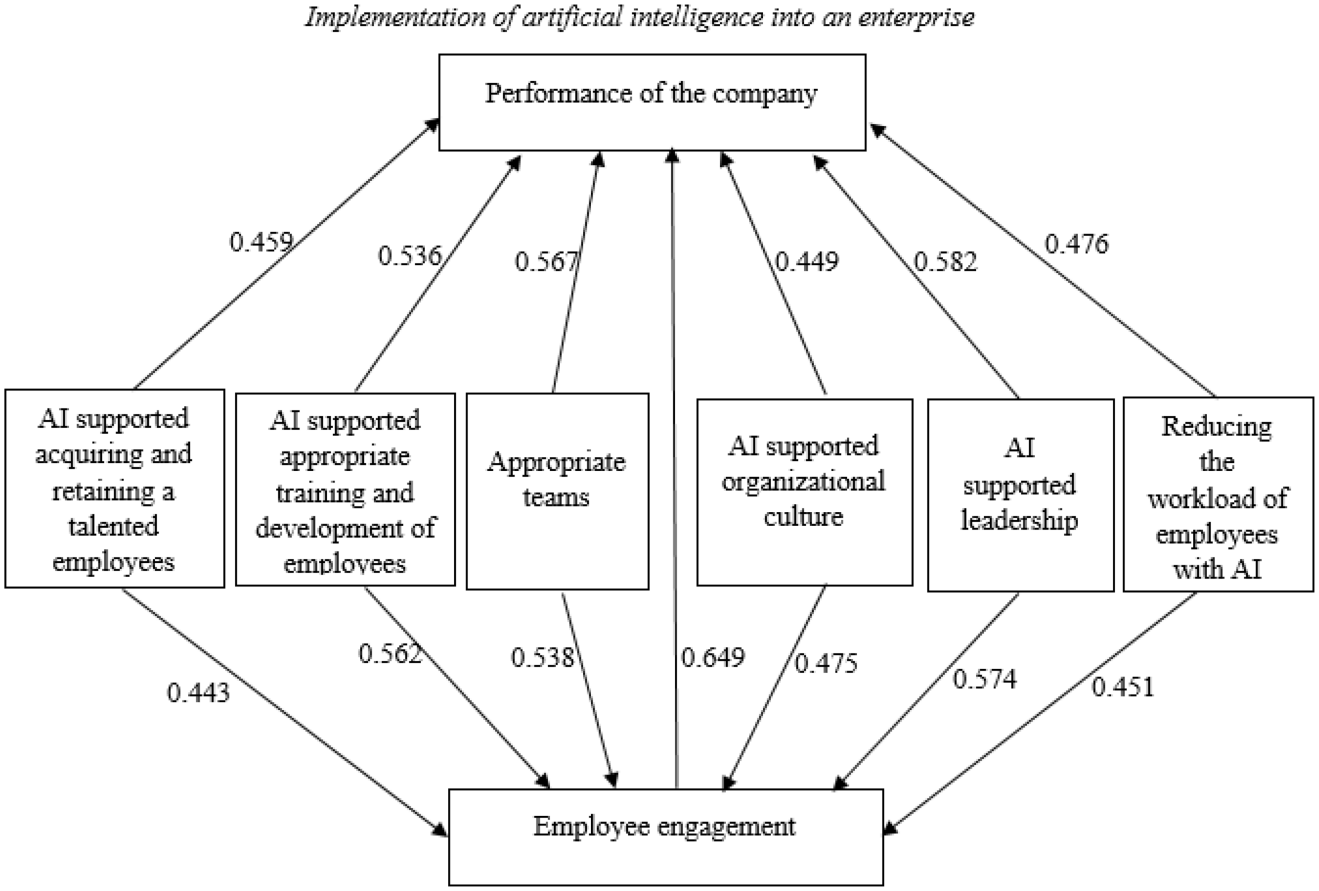
The conceptual model with the values of path coefficients.

Table 5 presents the results of SEM. Figure 2 presents the conceptual model with the values of path coefficients.

**Table 5:** Standardized path coefficients for the proposed model.

Hypothesized path	Path coefficient (γ)	Sig.	Effect size (ƒ^2^)	Standard error	Link direction	Shape of link
AR → PC	0.459	*p* < 0.05	0.354	0.031	Positive	Nonlinear
TD → PC	0.536	*p* < 0.05	0.412	0.031				
AT→PC	0.567	*p* < 0.01	0.389	0.030				
OC → PC	0.449	*p* < 0.001	0.352	0.031				
L → PC	0.582	*p* < 0.001	0.421	0.030				
RW → PC	0.476	*p* < 0.05	0.376	0.032				
AR → EE	0.443	*p* < 0.05	0.357	0.031				
TD → EE	0.562	*p* < 0.05	0.459	0.030				
AT→EE	0.538	*p* < 0.01	0.395	0.030				
OC → EE	0.475	*p* < 0.01	0.356	0.032				
L → EE	0.574	*p* < 0.001	0.465	0.031				
RW → EE	0.451	*p* < 0.01	0.353	0.031				
EE → PC	0.649	*p* < 0.01	0.517	0.029				

AR, acquiring and retaining a talented employees; TD, appropriate training and development of employees; AT, appropriate teams; L, leadership; RW, reducing the workload of employees; PC, performance of the company; EE, Employee engagement.

The results in Table 5 and Figure 2 show that AI supported acquiring and retaining a talented employees (AR → PC = 0.459, *p* < 0.05; AR → EE = 0.443, *p* < 0.05), AI supported appropriate training and development of employees (TD → PC = 0.536, *p* < 0.05; TD → EE = 0.562, *p* < 0.05), appropriate teams (AT→PC = 0.567, *p* < 0.01; AT→EE = 0.538, *p* < 0.01), AI supported organizational culture (OC → PC = 0.449, *p* < 0.001; OC → EE = 0.475, *p* < 0.01), AI supported leadership (L → PC = 0.582, *p* < 0.001; L → EE = 0.574, *p* < 0.001) and reducing the workload of employees with AI (RW → PC = 0.476, *p* < 0.05; RW → EE = 0.451, *p* < 0.01) have an effect on performance of the enterprise and employee engagement. Also, employee engagement has an effect on performance of the enterprise (EE → PC = 0.649, *p* < 0.01). Thus, we confirmed hypotheses H1–H13.

## Discussion

Table 5 and Figure 2 show that AI supported acquiring and retaining talented employees have a positive effect on performance of the enterprise and employee engagement. AI supported appropriate training and development of employees have a positive effect on performance of the enterprise and work engagement in Slovenian enterprises. Table 2 shows that the most important role in AI supported acquiring and retaining talented employees is employing candidates who have the appropriate skills to perform their work successfully. Nowadays, it is extremely important for the success of an enterprise to select the right candidates for the work. AI helps enterprises avoid the bias that always occurs in the recruitment process when employers decide on candidates. In addition, artificial intelligence can help identify opportunities for growth in existing employees, training needs, and further advancement (Lee and Chen, 2022). Artificial intelligence can connect and make connections between employee development opportunities and the arrival of new employees, leading to higher employee engagement (Di Francescomarino and Maggi, 2020). Lack of career advancement opportunities is one of the common reasons for decreased employee engagement and why employees decide to leave (Chiarini et al., 2020). Therefore, the enterprise should provide continuous learning programs such as supplementary skills training and subsidies for seminars. Have a solid career development and promotions system as part of the enterprise benefits. It is also imperative to make sure that the enterprise is able to adapt to certain changes in the industry and the overall business landscape. According to Spartaq. E-learning (2022), using artificial intelligence tools for employee education increases their productivity by 30%. While learning with artificial intelligence tools, employee engagement is 18% better than traditional methods, reducing the time required to learn by 65%.

Table 5 and Figure 2 show that appropriate teams have a positive effect on performance of the enterprise and employee engagement. Table 2 shows that the most important role of appropriate teams is that all team members achieve their goals equally effectively, followed by the team members producing many novel and valuable ideas. According to Arslan et al. (2021), successful teams are connected, accept diversity, and know how to find a common language. In this way, the teams will focus on different views on solving and achieving the enterprise’s successful goals (Webber et al., 2019; Arslan et al., 2021). Therefore, for a successful team, it is necessary to select individuals with different expertise and personality types that complement each other. Successful artificial intelligence teams also exhibit empathy for customers and other users. This ultimately paves the way for solving problems holistically. Understanding the problem to depth makes the individuals creative, curious, and innovative beyond imagination. According to Shaikh and Cruz (2022), for a team to be productive and effective, its members must be united by the same vision and committed to bring that visionto life (Shaikh and Cruz, 2022).

Moreover, for an enterprise to become ready for the future, its leaders must create an innovative organizational culture (Behl et al., 2021). Organizational culture is key to building an artificial intelligence-driven enterprise (Munir et al., 2022). The enterprises that manage to build a positive artificial intelligence culture and an inclusive and inspiring environment will successfully manage change and attract all their employees with artificial intelligence teams (Behl et al., 2021; Jarrahi et al., 2022). This is in line with our research findings that AI supported organizational culture has a positive effect on employee engagement and performance of the enterprise. Table 2 shows that the most important role of AI supported organizational culture is that policies of the enterprise are clearly defined, followed by employees fully understanding the enterprise’s goals. The third important role of AI supported organizational culture is that employees are familiar with all the services/ products that offer/produce in an enterprise, followed by using AI technology in any part of business and the enterprise’s culture is very responsive and changes easily. The average level of agreement with the statement “we used AI technology in any part of our business” is 3.58, which means that employees on average agree but do not completely agree. The results of the survey show that enterprises are embarking on the implementation of artificial intelligence and changing their organizational culture to embrace AI, but the average value of agreement is still low. In the modern economy, data is an invaluable resource in any business. AI is effective at quickly processing data to generate relevant answers to any questions that arise in business. Thus, the main aim of leaders is to create an organizational culture that will allow the organization to quickly develop and adapt to new business realities.

Table 5 and Figure 2 show that AI supported leadership has a positive effect on performance of the enterprise and on employee engagement. Table 2 shows that the most important role of AI supported leadership is to developed a clear vision for what was going to be achieved by department, followed by strong leadership to support artificial intelligence initiatives. According to Wang (2021), leaders are an essential part of any enterprise’s success. Leaders provide the vision that drives other enterprise employees to realize their goals (Niehueser and Boak, 2020; Wang, 2021). Technology has strongly interfered with existing ways of working, especially in routine and repetitive tasks. The trend will only intensify with the development and application of artificial intelligence, which will greatly change leaders’ work (Wijayati et al., 2022). Today, the leaders need to inculcate the right skills to help enterprises maintain a sense of competitiveness in aspects of upskilling as well as initiating mentoring for the betterment of the teams.

Table 5 and Figure 2 show that reducing the workload of employees has a positive effect on employee engagement and performance of the enterprise. Table 2 shows that the most important role of reducing employees’ workload with AI is to reduce the burden on administrative staff with artificial intelligence in the enterprise, followed by artificial intelligence technology applied can communicate with users/customers, which reduces the workload of employees. Reducing employee workloads can have a tremendous impact on stress levels and free up their schedules to focus on tasks that artificial intelligence cannot automate. According to a survey of 34.000 employees in 18 countries, 72% of working professionals attribute low stress levels to productivity boosting tools and tech. Employees are welcoming artificial intelligence-powered automation technology as it will help them work more effectively and reduce stress. Thus, the implementation of artificial intelligence into an enterprise has a positive effect on performance of the enterprise. Enterprises that have adopted artificial intelligence in their operations have seen great success (Palanivelu and Vasanthi, 2020). Table 2 shows that the most important role of performance of the enterprise is the profitability and innovativeness of the enterprise. Following, artificial intelligence accelerates making quick and better decisions to achieve successful results. According to Bag et al. (2020), Chiarini et al. (2020), Lezoche et al. (2020), and Wamba-Taguimdje et al. (2020), as enterprises are becoming more employee-centric, artificial intelligence is helping them create a happier work environment, increase workforce’s productivity and creating a positive experience which lead to higher employee engagement and performance of the enterprise.

### Theoretical and managerial implications

The implementation of artificial intelligence in an enterprise is a comprehensive change of the enterprise’s processes and may lead to greater productivity, growth and competitiveness of the enterprise. The use of AI technology offers new business opportunities to enterprises, greater productivity, new ways of designing business models of enterprises, encourages innovation and development, and new ways of promoting. From this point of view, we have developed a multidimensional talent management model with embedded aspects of artificial intelligence in the human resource processes to increase employees’ engagement and performance of the enterprise. The research model highlights the importance of certain aspects that are necessary for the successful operation of an enterprise in today’s rapidly changing environment. Thus, regardless of the size of the enterprise, the following aspects must be taken into account when implementing artificial intelligence to increase performance of the enterprise and employee engagement: AI supported acquiring and retaining talented employees, AI supported appropriate training and development of employees, appropriate teams, AI supported organizational culture, AI supported leadership and reducing the workload of employees with AI. The development of a digital society and the digitization of the economy requires appropriate legislation and an environment that will encourage the development of digitization and digital entrepreneurship. It is necessary to facilitate access to financial resources for enterprises, especially for SMEs, so that they can more easily access funds for financial investment in technological development, digital transformation, artificial intelligence, knowledge and skills, which will help them to be more competitive in Slovenia and in the international business environment.

### Limitations and further research

Our study is limited to a sample of managers/owners in Slovenian enterprises. Limitations of our research are reflected in the size of enterprises because we selected medium-sized and large enterprises. The results show the situation of medium-sized and large enterprises and do not provide conclusions for small enterprises, especially in terms of whether the use of AI is suitable for small enterprises. The challenge faced by micro, small and medium-sized enterprises (SMEs) is certainly the fear of changes brought about by digitization and digital transformation of enterprises. When dealing with challenges, SMEs often have insufficient information, financial resources and personnel who have the appropriate skills for the digital transformation of the enterprise. The limitations of our research are also reflected in the constructs that we have chosen for the survey. Therefore, we recommend further research to develop new constructs for example, implementation of management information system into the enterprise, adopting AI technologies, using AI solutions in a project, enterprise’s competitiveness and analyze them through structural equation modeling. Also, for further research, we suggest the examination of constructs that we have chosen for the survey in other countries to compare the results.

## Author’s note

Artificial intelligence has taken over the enterprise sector and changed the way enterprises do business today. Thus, the new advancing trends like artificial intelligence and other cutting-edge technologies are transforming the way of work, changing the workforce’s profile. The paper highlights the important constructs for successfully implementing artificial intelligence applications in the enterprise to increase employee engagement and performance of the enterprise. The main highlights that enterprises need to be aware of when implementing artificial intelligence are improving employee engagement and productivity, gaining knowledge in the field of artificial intelligence, and increasing an enterprise’s profitability. Artificial intelligence is becoming part of the digitization strategy as it enables enterprises to leverage vast amounts of process data with advanced algorithms to improve efficiency and reduce production costs. The use of artificial intelligence in production will be especially increased for quality control. Artificial intelligence is very promising for visual inspection of parts at different stages of the product development process and in all workplaces, including production and assembly, with the aim of achieving a certain quality. As the demand for innovative products increases rapidly, the use of artificial intelligence is becoming essential to understanding customer needs, requirements, and desires.

## Data availability statement

The original contributions presented in the study are included in the article/supplementary material, further inquiries can be directed to the corresponding author.

## Ethics statement

Ethical review and approval was not required for the study on human participants in accordance with the local legislation and institutional requirements. Written informed consent from the patients/participants or patients/participants legal guardian/next of kin was not required to participate in this study in accordance with the national legislation and the institutional requirements.

## Author contributions

All authors listed have made a substantial, direct, and intellectual contribution to the work and approved it for publication.

## Funding

This work has been fully supported by Croatian Science Foundation under the project UIP-2020-02-6312. The authors acknowledge the financial support from the Slovenian Research Agency (research core funding No. P5–0023, ‘Entrepreneurship for Innovative Society).

## Conflict of interest

The authors declare that the research was conducted in the absence of any commercial or financial relationships that could be construed as a potential conflict of interest.

## Publisher’s note

All claims expressed in this article are solely those of the authors and do not necessarily represent those of their affiliated organizations, or those of the publisher, the editors and the reviewers. Any product that may be evaluated in this article, or claim that may be made by its manufacturer, is not guaranteed or endorsed by the publisher.
